# Protocol deviations in medicine quality tests

**DOI:** 10.2471/BLT.24.292994

**Published:** 2025-10-23

**Authors:** Esti Mulatsari, Ismail Dwi Saputro, Ronal Simanjuntak, Vicky Achmad Ginanjar, Ayu Rahmawati, Elizabeth Pisani, Yusi Anggriani

**Affiliations:** a Undergraduate Program, Faculty of Pharmacy, Pancasila University, Jl. Srengseng Sawah, RT.5/RW.5, Srengseng Sawah, Kec. Jagakarsa South Jakarta City, Jakarta 12630, Indonesia.; b PT. Equilab International Laboratory, Jakarta, Indonesia.; c Centre for Pharmaceutical Policy, Pancasila University, Jakarta, Indonesia.

## Abstract

National, regional and international pharmacopoeias set standards for the quality of medicines and provide instructions for testing to ensure those standards are met. Medicines, tests, standards and methods all vary among the more than 60 pharmacopoeias currently in use. These publications were developed mainly to regulate medicine manufacturing, where safety is essential and methods are generally complex. These methods require sophisticated equipment, specific materials and high levels of skill, and can generate large quantities of pharmaceutical waste. However, pharmacopoeias are also used to guide medicine quality testing for other purposes and in other settings. Notably, they are used in post-marketing surveillance and research on medicine quality in low- and middle-income countries where the prevalence of substandard and falsified medicines is thought to be highest. Regulators and other researchers in these settings may not have access to all the equipment, materials and skills needed to follow pharmacopoeias exactly. Therefore, they often develop work-arounds, which are rarely acknowledged or described when reporting results. Our experience using modified methods for testing amoxicillin tablets in Indonesia suggests that necessary work-arounds can significantly distort study outcomes, potentially leading to misguided policy responses. We argue that standard-setting bodies should recognize challenges to testing in research and surveillance contexts in resource-constrained settings where patient safety is not at immediate risk. These bodies should provide evidence-based guidance on low-cost, environmentally sustainable modifications to industry-standard testing methods for use in these contexts. The research community must inform this guidance, providing details of modifications and their outcomes, both successful and unsuccessful.

## Résumé

Les pharmacopées nationales, régionales et internationales fixent des normes de qualité pour les médicaments et fournissent des instructions pour les tests visant à garantir le respect de ces normes. Les médicaments, tests, normes et méthodes varient d’une pharmacopée à l’autre, parmi les plus de 60 pharmacopées actuellement en usage. Ces instructions ont été élaborées principalement pour réglementer la fabrication des médicaments, où la sécurité est essentielle et les méthodes sont généralement complexes. Celles-ci nécessitent des équipements sophistiqués, des matériaux spécifiques et un niveau élevé de compétence et peuvent générer de grandes quantités de déchets pharmaceutiques. Cependant, les pharmacopées servent également à orienter les tests de qualité des médicaments à d’autres fins et dans d’autres contextes, notamment dans la surveillance postérieure à la mise sur le marché et la recherche sur la qualité des médicaments dans les pays à revenu faible et intermédiaire, qui connaissent vraisemblablement le taux le plus élevé de médicaments falsifiés et de qualité inférieure. Les régulateurs et autres chercheurs travaillant dans ces conditions peuvent ne pas avoir accès à tous les équipements, matériaux et compétences nécessaires pour appliquer à la lettre les pharmacopées. Ils mettent donc souvent au point des solutions de contournement, rarement reconnues ou décrites lorsqu’ils publient des résultats. Notre expérience en matière d’utilisation de méthodes modifiées pour tester des comprimés d’amoxicilline en Indonésie suggère que les solutions de contournement nécessaires sont de nature à fausser considérablement les résultats des études, ce qui peut conduire à des réponses politiques inappropriées. Les organes de normalisation devraient selon nous reconnaître les difficultés liées aux tests dans la recherche et la surveillance dans des contextes de ressources limitées et d’absence de menace immédiate pour la sécurité des patients. Ils devraient également formuler des recommandations fondées sur des données probantes concernant des modifications peu coûteuses et durables sur le plan environnemental des méthodes de test standard du secteur pharmaceutique à utiliser dans ces contextes. La communauté scientifique doit contribuer à l’élaboration de ces recommandations en fournissant des détails sur les modifications et leurs résultats, qu’ils soient positifs ou négatifs.

## Resumen

Las farmacopeas nacionales, regionales e internacionales establecen normas para garantizar la calidad de los medicamentos y proporcionan instrucciones para realizar las pruebas que aseguren el cumplimiento de esas normas. Los medicamentos, las pruebas, las normas y los métodos varían entre las más de 60 farmacopeas actualmente en uso. Estas publicaciones se desarrollaron principalmente para regular la fabricación de medicamentos, ámbito en el que la seguridad es esencial y los métodos suelen ser complejos. Estos métodos requieren equipos sofisticados, materiales específicos y un alto nivel de competencia técnica, además de generar grandes cantidades de residuos farmacéuticos. Sin embargo, las farmacopeas también se utilizan como referencia para las pruebas de calidad de los medicamentos con otros fines y en diferentes contextos. En particular, se emplean en la vigilancia posterior a la comercialización y en la investigación sobre la calidad de los medicamentos en países de ingresos bajos y medios, donde se considera que la prevalencia de medicamentos subestándar y falsificados es mayor. En estos entornos, los organismos reguladores y otros investigadores pueden no disponer de todos los equipos, materiales y competencias necesarios para aplicar las farmacopeas con exactitud. Por ello, suelen desarrollar soluciones alternativas, que rara vez se reconocen o describen al comunicar los resultados. La experiencia en el uso de métodos modificados para analizar comprimidos de amoxicilina en Indonesia sugiere que estas soluciones pueden distorsionar significativamente los resultados de los estudios, lo que podría dar lugar a respuestas normativas inadecuadas. Los organismos encargados de establecer normas deberían reconocer los desafíos que plantea la realización de pruebas en contextos de investigación y vigilancia en entornos con recursos limitados, donde la seguridad del paciente no se ve amenazada de forma inmediata. Estos organismos deberían proporcionar orientaciones basadas en la evidencia sobre modificaciones de bajo costo y sostenibles desde el punto de vista ambiental de los métodos normalizados de la industria para su uso en dichos contextos. La comunidad científica debe contribuir a la elaboración de estas orientaciones, al proporcionar información detallada sobre las modificaciones y sus resultados, tanto exitosos como fallidos.

## ملخص

تضع دساتير الأدوية الوطنية والإقليمية والدولية معايير لجودة الأدوية، وتوفر تعليماتٍ للاختبار لضمان الوفاء بها. وتختلف جميع الأدوية، والاختبارات، والمعايير القياسية، والطرق بين أكثر من 60 دستورًا دوائيًا مستخدمًا حاليًا. وقد طُوّرت هذه المنشورات بشكل رئيسي لتنظيم تصنيع الأدوية، حيث تُعدّ السلامة أمرًا بالغ الأهمية، وتكون الأساليب معقدة بشكل عام. تتطلب هذه الأساليب معداتٍ متطورةً، وموادًا محددة، ومهاراتٍ عالية، ويمكن أن تنتج كمياتٍ كبيرةً من النفايات الدوائية. ومع ذلك، تُستخدم دساتير الأدوية أيضًا لتوجيه اختبارات جودة الأدوية لأغراضٍ أخرى وفي سياقاتٍ أخرى. والجدير بالذكر أنها تُستخدم في مراقبة ما بعد التسويق، وفي أبحاث جودة الأدوية في الدول ذات الدخل المنخفض والدخل المتوسطة، حيث يُعتقد أن انتشار الأدوية دون المستوى المطلوب والمغشوشة هو الأعلى. قد لا يتمكن المنظمون والباحثون الآخرون في هذه السياقات من الوصول إلى جميع المعدات، والمواد، والمهارات اللازمة لاتباع دساتير الأدوية بدقة. لذلك، غالبًا ما يطورون حلولًا بديلة، ونادرًا ما يتم الإقرار بها أو وصفها عند الإبلاغ عن النتائج. تشير تجربتنا في استخدام أساليب مُعدّلة لاختبار أقراص أموكسيسيلين في إندونيسيا إلى أن الحلول البديلة الضرورية يمكنها أن تُشوّه نتائج الدراسات بشكل كبير، مما قد يؤدي إلى استجابات مُضلّلة للسياسة. نحن نؤكد على ضرورة أن تدرك هيئات وضع المعايير التحديات التي تواجه الاختبارات في سياقات البحث والمراقبة في البيئات محدودة الموارد، حيث لا تُشكّل سلامة المرضى خطرًا داهمًا. يجب على هذه الهيئات تقديم إرشادات قائمة على الأدلة حول التعديلات منخفضة التكلفة، والمستدامة بيئيًا، على أساليب الاختبار القياسية في هذه الأوضاع. يجب على مجتمع البحث إبلاغ هذه الإرشادات، وتقديم تفاصيل عن التعديلات ونتائجها، سواءً كانت ناجحة أو غير ناجحة.

## 摘要

国家、地区和国际药典均列明了药品质量相关标准并提供了检验说明，以确保严格遵循此类标准。现行有效的 60 多部药典所列药品、检验项目、质量标准和检验方法均各不相同。发行此类出版物的主要目的在于监管药品制造，因为在药品制造领域，安全性至关重要，而检验方法通常比较复杂。实施此类检验方法需要具备精密设备、特定材料和高超的技能水平，且可能会产生大量医药废物。然而，药典还可用于指导出于其他目的和在其他场景下进行的药品质量检验。特别是在被公认为伪劣药品泛滥最为严重的中低收入国家，药典通常被用于上市后监测和药品质量研究。在这些场景下，监管者和其他研究人员可能无法获得完全符合药典要求的所有设备、材料和技能。因此，他们通常会采取变通方法，但他们在汇报结果时很少会承认或说明此点。我们在印度尼西亚采用改良方法对阿莫西林片进行检验的经验表明，在迫不得已的情况下采取的变通方法可能会导致研究结果严重失真，从而可能会致使引发错误的政策反应。我们认为，标准制订机构应认识到在资源有限的情况下出于研究和监测目的开展检验工作（这并不会直接威胁到患者安全）时需面临的挑战。此类机构应提供循证指南，说明如何以低成本且环境上可持续的方式对行业标准检验方法进行修改，从而使其适用于此类场景。研究团体必须深入了解此类指南，并详细说明其对检验方法做出了哪些修改以及修改后产生的效果（无论成功与否）。

## Резюме

Национальные, региональные и международные фармакопеи устанавливают стандарты качества лекарственных средств и дают указания по проведению испытаний для обеспечения соблюдения этих стандартов. Лекарственные средства, испытания, стандарты и методы различаются во всех используемых в настоящее время фармакопеях (их более 60). Эти публикации были в основном разработаны для регулирования производства лекарственных средств, где безопасность крайне важна и применяются в целом сложные методы. Эти методы требуют использования высокотехнологичного оборудования, специализированных материалов и высокой квалификации персонала и могут приводить к образованию значительного количества фармацевтических отходов. Тем не менее фармакопеи также используются как рекомендации для испытаний качества лекарственных средств для других целей и в других обстоятельствах. Среди таких случаев стоит отметить использование в послерегистрационном надзоре и в исследованиях качества лекарственных средств в странах с низким и средним уровнем доходов, где, как предполагается, лекарственные средства недостаточного качества и фальсифицированные препараты встречаются наиболее часто. В этих условиях регулирующие органы и прочие исследователи могут не иметь доступа ко всему оборудованию, материалам и специалистам, необходимым для точного соблюдения требований фармакопеи. Поэтому они зачастую разрабатывают обходные пути, наличие которых редко признается и которые не описываются при сообщении результатов. Наш опыт использования модифицированных методов для испытаний таблеток амоксициллина в Индонезии позволяет предположить, что такие вынужденные обходные способы могут существенно исказить результаты исследований, что в итоге может приводить к неправильному реагированию на уровне политик. Авторы постулируют, что органы, устанавливающие стандарты, должны учитывать наличие проблем с испытаниями в контекстах исследований и надзора в условиях ограниченных ресурсов в тех случаях, когда безопасность пациентов не подвергается непосредственному риску. Эти органы должны предоставлять опирающиеся на доказательства рекомендации по недорогим, экологически безопасным модификациям стандартных отраслевых методов испытаний для использования в указанных контекстах. Исследовательские сообщества должны предоставлять информацию для таких рекомендаций, сообщая подробные данные относительно модификаций и их результатов, как успешных, так и неудачных.

## Introduction

Testing medicines to ensure that they are safe is complicated and expensive. Quality standards are provided in the *International pharmacopoeia* published by the World Health Organization (WHO), as well as in more than 60 other national and regional pharmacopoeias.^1^^,^^2^ These documents also provide monographs detailing how to perform chemical and other testing for specific medicines. However, they may differ in the medicines they cover, use different definitions of what constitutes an in-specification medicine (that is, have different specifications for setting quality standards), and stipulate different equipment and methods for preparing and testing samples of the same medicine, formulation and dose.^3^ In addition, methods can be inflexible on some points, while remaining vague on others.

Medicine manufacturing presupposes access to capital, sophisticated equipment, good technical skills and an exact knowledge of a medicine’s contents and formulation. Manufacturers can thus be expected to have access to the resources they need to use pharmacopoeias effectively to ensure their products meet approved quality standards. In countries with substantial pharmaceutical manufacturing, national medicine regulators should also have the capacity to verify that producers are meeting agreed pharmacopoeial standards. However, these documents are also used in other settings. Some regulators sample medicines from retail outlets and health-care settings to check their quality; these may include products that are imported through special access schemes or illegally, for which the regulator does not have full formulation details.^4^ As international concern about exposure to substandard and falsified medicines grows, other researchers are increasingly conducting market surveys to assess the prevalence of poor quality medicines, particularly in lower-income settings were the prevalence is believed to be highest.^5^^,^^6^

In this paper, we focus on the testing challenges faced by researchers or institutions conducting surveys of medicine quality in resource-constrained settings and the implications these challenges may have for our understanding of threats to public health. We draw on our own experience assessing medicine quality in Indonesia and provide a case study of testing amoxicillin 500 mg tablets.

## Challenges

Although researchers around the world may face the challenges described below, they are most acute for researchers working in low- and middle-income settings, where access to funding, expertise and adequate staff time is often limited.

### Monographs and standards

Some pharmacopoeias charge for use of their monographs as well as use of the chemical standards required in testing. Best known among these publications are those of the *United States Pharmacopeia*, used in 140 countries and integrated into the laws of more than 40 countries.^7^ The *United States Pharmacopeia* charges 950 United States dollars (US$) a year for a subscription to its monographs for a single user, while its European equivalent costs 570 euros (€; US$ 645).^8^^,^^9^ Some other pharmacopoeias, including the *International Pharmacopeia*, are available free of charge. However, they are not always comprehensive.

In the *International Pharmacopeia* published by WHO, for example, monographs are not available for tablet forms of three of the five common essential medicines that were included in our Indonesian study: amoxicillin, amlodipine and cefixime.^7^ In addition, some essential tests may not be covered. The *International Pharmacopeia* monograph for allopurinol, for example, provides guidance for testing identity and measuring assay (that is, the percentage of labelled active pharmaceutical ingredients present). However, it does not include dissolution, which is a proxy for how a tablet might perform in the body.

The cost of reference standards also varies considerably. At regular prices in 2025, 200 mg of the primary reference standard for amoxicillin cost US$ 283 from the *United States Pharmacopeia*, €79 (US$ 90) from the *European Pharmacopeia* and 500 000 Indonesian rupiah (US$ 30) from Indonesia’s Infalabs, a service provided by the national medicine regulator.^10^^–^^12^ Academic discounts are sometimes available; and cheaper, higher volume secondary reference standards, recognized by some of the more widely used pharmacopoeia, are also available for many commonly tested active ingredients.^13^ However, access to monographs and standards can still stretch budgets.

### Equipment and materials

Monographs vary greatly in the specificity with which they describe both the methods and equipment necessary for compendial tests such as high-performance liquid chromatography. The more specific the monographs are about equipment, the harder it may be to comply precisely with their requirements in settings where access to pieces of equipment meeting particular specifications is limited.

In addition, to obtain representative sample solutions, many monographs prescribe methods that use large quantities of sometimes costly solvents or other substances, but ultimately only tiny amounts of the solutions are used in testing. This situation increases costs and poses a challenge of waste disposal, especially when the unused product is toxic, environmentally harmful or contributes to drug resistance.

### Validation and verification

Although many monographs leave room for interpretation, using vague phrases such as “transfer to a suitable volumetric flask, dilute with water to [unspecified] volume", the methods in monographs are considered to have been validated. Laboratories need to perform verification tests to ensure that their equipment is operating as required, but if they follow the monograph, they do not need to repeat full validation, which ensures that a test, conducted as specified, yields a valid result. Full validation is also not needed if a laboratory’s deviation from chromatographic conditions (e.g. adjustments to column dimensions, flow rate, column temperature and salt concentrations) falls within specified limits.^14^ When the equipment, materials, human resources or time needed to follow a monograph precisely are not available, laboratory staff may modify procedures, for example using an alternative solvent or a different filtration method. To eliminate any need for revalidation of methods, such modifications are best avoided. However, practical work-arounds are a fact of life in resource-constrained settings. Where such work-arounds are necessary, pharmacopoeias generally require that they be validated before use. The *United States Pharmacopeia* and guidance from the International Council for Harmonisation of Technical Requirements for Pharmaceuticals for Human Use on validation of compendial procedures also state that validation is needed when there are changes in the composition of a drug product.^15^^,^^16^

However, performing this validation may be challenging. First, validations are best undertaken in collaboration with multiple independent laboratories accredited in testing the specific medicine formulations, for example through accreditation by the International Organization for Standardization or WHO laboratory prequalification. In practice, few low- and middle-income countries have access to any laboratory accredited for medicine quality testing; where these countries do have access, these facilities are often run by regulatory institutions with limited capacity to take on external validation work.

Second, validation is a time-consuming, multistep process requiring high levels of chemistry skills, which are typically in short supply in resource-constrained settings.

Third, market surveys often sample many different medicines and brands. Even regulators may sample unauthorized products with unknown excipients (substances other than active pharmaceutical ingredients in finished pharmaceutical products), and academic researchers rarely know the precise formulation of each product. Since different manufacturers use different excipients, compliance with guidance given by the *United States Pharmacopeia* and the International Council for Harmonisation on validation requires a new validation procedure for every different dosage and brand. Even if the skills were available, neither research nor surveillance budgets allow for this work.

Finally, validation work may consume samples and expensive standards. Samples in our study cost around US$ 147 each to collect and process, before any laboratory costs.^17^ Using samples and reference chemicals for validation is an additional cost. Furthermore, when samples collected as part of a field survey are used to validate methods rather than contribute to study results, the representativeness of surveys based on random sampling designs is compromised.

### Expertise

Different modifications of the methods will have different outcomes, as our case study described later illustrates. However, in resource-constrained settings, laboratory staff may have limited expertise or experience in testing specific pharmaceutical samples and are unlikely to know the full composition of a product. Guidelines of the International Conference of Harmonisation recognize the challenges of revalidation described in the previous section, and recommend that analysts use their judgement: “Science and risk-based principles can be used to justify whether or not a given performance characteristic needs revalidation".^16^ But less-experienced laboratory staff may have difficulty determining which parts of a mandated procedure can most readily be modified to minimize potential distortion to testing results. Pharmacopoeias do not currently provide detailed information that would support such decision-making.

### Real-world example, Indonesia

Our Jakarta-based academic research group experienced many of the challenges described above while implementing STARmeds, a large study of the quality of five medicines collected online and from randomly selected retail outlets in Indonesia. The STARmeds study methods and results are reported in detail elsewhere.^18^

Here we give a brief summary of a case study of amoxicillin testing. All of the procedures are reported in detail in the online repository.^19^ During the study, we collected 231 samples of amoxicillin 500 mg tablets from 32 different brands and another seven distinct manufacturer-specific unbranded generic versions. 

The ISO/IEC 17025-certified private laboratory that tested the samples used the *United States Pharmacopeia* reference standards and the *United States Pharmacopeia 43-National Formulary 38* monograph for amoxicillin tablets,^18^ but was unable to source a specific flask size required by the monograph. After considering data on the solubility of amoxicillin trihydrate, the laboratory therefore modified the procedure. In this modification, five tablets were dissolved in a smaller flask first and then the resulting solution was further diluted to match the final concentration of the active pharmaceutical ingredient required by the *United States Pharmacopeia* monograph (1 mg/mL). The laboratory and STARmeds staff judged that the deviation from the validated *United States Pharmacopeia *method was not large enough to require revalidation.

We tested 75 samples thus prepared using high-performance liquid chromatography. In 40 cases, the amoxicillin tablets contained less than the minimum permitted amount of labelled active pharmaceutical ingredient (90.0%), a 53.3% failure rate. This rate was more than 90 times higher than the failure rate reported for 1365 amoxicillin samples collected in random and risk-based sampling by the Indonesian medicine regulator (Indonesian Food and Drug Authority) between 2018 and 2020 (data shared by the regulator with the STARmeds research team).

Given this discrepancy, we paused testing, conducted a laboratory investigation and concluded that the two-step sample solution (online repository)^19^ was the most likely source of the high failure rate.

**Fig. 1:**
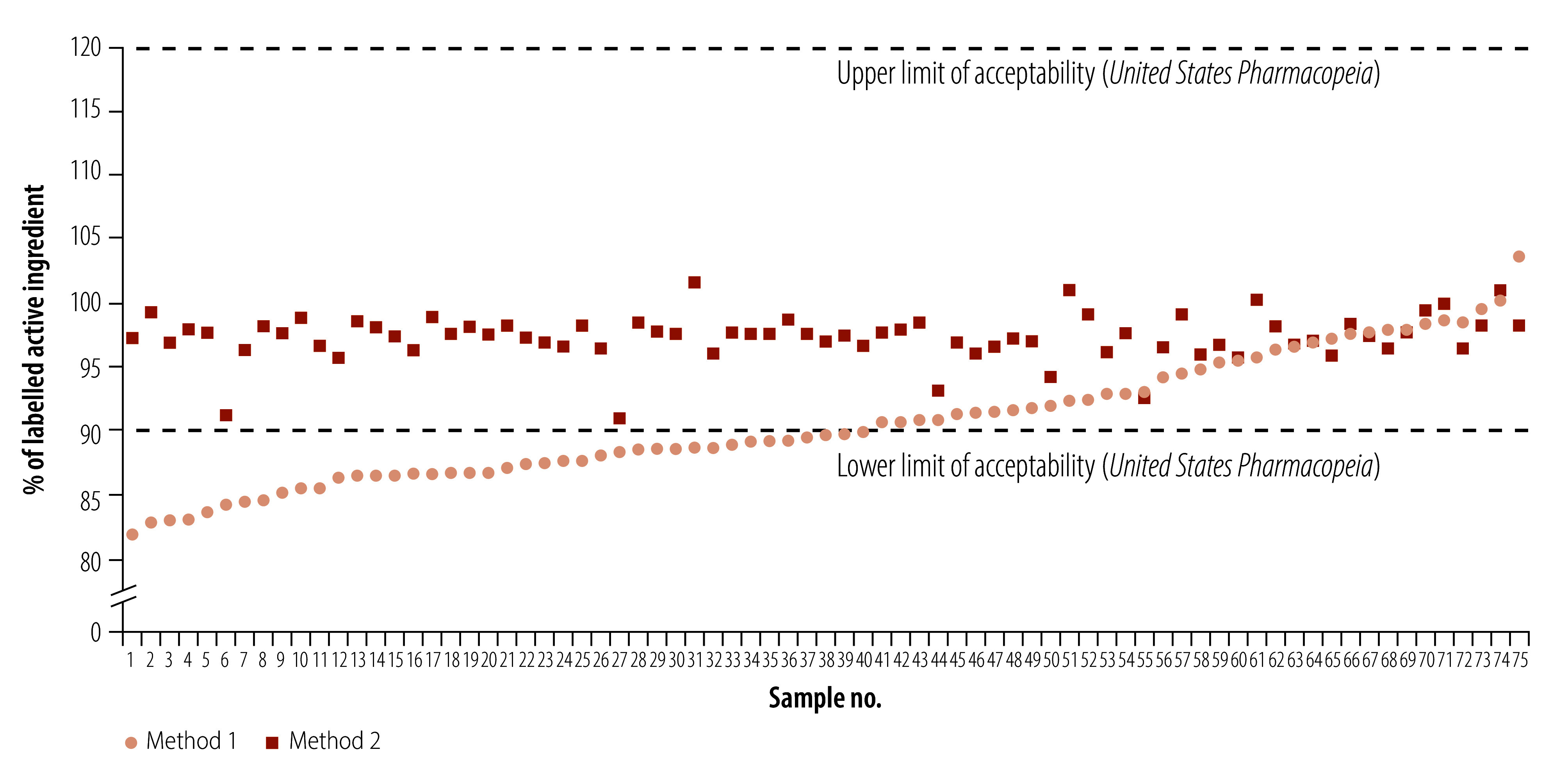
Chemical assay testing of amoxicillin 500 mg using two methods of preparation of the sample solution

Note: the two methods are described in the section: Real-world example, Indonesia.

Since the flasks needed to comply exactly with the *United States Pharmacopeia* monograph were still not available, we devised a second work-around. We split five tablets into three groups (2:2:1) and dissolved them in three separate flasks, and then mixed the resulting solution. This method at no point exceeded the recommended 1 mg/mL concentration and is much closer to that used in the *United States Pharmacopeia* monograph. We retested all 75 previously processed samples using this second method. As Fig. 1 shows, more than half of the samples fell below the permitted threshold of 90% active ingredient using the first method. However, when retested using the second method, all samples were above the threshold, and thus complied with the quality standard.

The results achieved using method 2 matched far more closely the prevalence of substandard amoxicillin reported by the Indonesian Food and Drug Authority. The Authority’s laboratory has been pre-qualified by WHO for medicine quality testing since 2019 and we consulted them during our investigation. We used the second method for all of the remaining amoxicillin tablets tested in the study. A total of 15/188 amoxicillin tablets failed testing using the second preparation method, giving an unweighted prevalence of substandard samples of 8.0%. When weighted by the market share of each brand, the prevalence fell to an estimated 6.3%.^18^

## Implications

The most obvious lesson we draw is that variations in methods for medicine quality testing can have a substantial impact on reported results. And yet a search of the literature conducted as part of our laboratory investigation revealed that it was rare to find such variations acknowledged, and rarer still to find them reported in detail. Here, we offer additional reflections and recommendations to improve guidance.

### Prevalence of poor quality medicines

In 2017, WHO published a meta-analysis of 100 published field surveys of medicine quality, mostly in low- and lower middle-income countries. The headline, that one in 10 medicines in those countries was substandard or falsified, captured the public imagination, even though the report included many cautions about data quality.^5^^,^^20^ Absent from those warnings was any discussion of the possible effect of unreported deviations from validated testing procedures. Modified sample preparation methods are unlikely to find an active ingredient that is not there, but they can (as in our first modified preparation method) lead to incomplete dissolution and release that would overstate the prevalence of under-dosed medicines and the public health risks posed by such medicines. The risk may be especially important for antibiotics because under-dosing, as well as harming the patient, may increase the risks of antimicrobial resistance.^21^ Thus, studies on medicine quality that do not report sample preparation methods and other details of testing should be viewed with caution. Furthermore, reputable organizations should not repeat headline statistics drawn from those studies.

### Pharmacopoeia evidence

While different pharmacopoeias recommend different methods, materials and standards, they very rarely share the evidence underlying their recommendations. The acceptable range of the labelled active ingredient in the *United States Pharmacopeia 43-National Formulary 38* is 93–107%, whereas the range in the 12th edition of the International Pharmacopoeia is 90–110%. Thus, regulators using the *United States Pharmacopeia* standard would consider an allopurinol tablet containing 92% or 108% of the labelled active ingredient to be substandard, whereas regulators using the *International Pharmacopoeia* would consider both samples to be of acceptable quality. Why is there this difference? Neither monograph provides evidence for the reasoning behind their recommendations of these cut-offs.

The International conference of harmonisation and *United States Pharmacopeia* agree that method validation is required if there are changes in the composition of the drug product. However, most pharmacopoeias address only the active ingredient and say nothing about the excipients, which vary between manufacturers. This issue calls into question the very notion of a so-called prevalidated method for a finished formulation. Since users do not know the composition for which a method has been validated, they must in theory revalidate methods for every brand or manufacturer with non-identical or unknown formulations.

These contradictions, and the lack of transparency underlying them, create confusion, including about when validation is needed, and when and why products should be considered substandard. We recommend that the International council for harmonisation and WHO encourage publication of the evidence on which pharmacopoeia methods and standards are chosen and considered validated.

We propose that monographs provide information about the likely effect of different types of excipients on the accuracy of recommended methods. We also urge WHO to include robust, cost-effective and waste-limiting monographs for more of the essential medicines and formulations most commonly used in low- and middle-income countries, including amoxicillin and amlodipine tablets.

### Purpose of testing

Pharmacopeia standards were originally developed in rich countries, primarily to help regulators ensure that profit-driven manufacturers made medicines that did not harm patients. When used in this context, it is reasonable to expect those manufacturers to assume the costs of performing all necessary tests to the most rigorous standards, including validating methods for new formulations. This reasoning holds wherever the manufacturer is based. There is no reason why a pharmaceutical company in a low-income country that makes unbranded generic medicines on contract for other companies at home or abroad should be held to lower safety standards than multinationals manufacturing patented medicines in high-income settings; for producers, safety must always be paramount.

In the context of medicine quality surveys, including post-market surveillance by regulators in resource-constrained settings, however, this level of rigour may not be necessary. We simply need methods that are considered good enough: that is, feasible with locally available skills and resources, that yield results sensitive enough to trigger further investigation where necessary, and are specific enough to avoid creating excessive burdens for regulators. These methods should minimize expense and the creation of waste (such as the potentially unnecessary generation of about 2490 mL of unused antibiotic solution per single test of the 500 mg amoxicillin tablet sample tested), and align with the sustainability aspirations expressed at the 14th International meeting of the world pharmacopoeias.^22^

We recommend that standard-setting organizations, including WHO, provide guidance on specific contexts in which methods may be modified and extend monographs to include tried-and-tested modifications for those contexts.

### Experience sharing

Comparing the prevalence of substandard amoxicillin tablets in STARmeds with the results obtained by Indonesia’s WHO-prequalified regulatory laboratory (testing samples of the same brands collected in similar settings), we conclude that the sample preparation used in our first modification was not good enough to produce valid results; it likely greatly over-estimated the true proportion of under-dosed tablets. The second modification, although not fully validated, was more similar to the method prescribed by *United States Pharmacopeia* and gave a result that more closely matched results recorded in other studies^23^ and by the regulator. This method was probably good enough for a prevalence survey.

Other modifications would have been possible, for example, crushing tablets before dissolving them. A study in Togo found high variability in assay results for amoxicillin–clavulanic acid tablets collected in the country. Although the *United States Pharmacopeia* monograph instructs “dissolve with the aid of mechanical stirring” for this formulation, the authors of the study reported that thorough mechanical disintegration improved reproducibility of results.^24^ Other researchers in tropical settings report sonicating for longer than recommended to increase sample solubility. When testing the quality of amoxicillin capsules in Kenya, a study used the same initial concentration as our first modification (2.5 mg/mL). However, the Kenyan study assessed the contents of capsules, not tablets, thus used materials that were already powdered. The authors also reported sonicating for 10 minutes, twice as long as the monograph-recommended period of 5 minutes which we used.^25^ A study in Democratic Republic of the Congo sonicated each sample of the anti-malarial artemether lumefantrine for 2 hours rather than the 20 minutes recommended by the *International pharmacopeia*.^26^

In resource-constrained research settings, sample solution preparation procedures such as powdering tablets and weighing out active ingredient-equivalent masses could reduce the need for specific flask sizes. A study in Lebanon suggested sampling techniques for powdered material designed to minimize the risk of introducing bias with these methods.^27^

These powdering techniques would also reduce consumption of solvent and generation of antibiotic waste, thereby saving money and reducing environmental and health-system impact. The studies cited here are unusual in that they provide details about modifications in sample preparation. However, informal discussions with colleagues in the field suggest that laboratory work-arounds are common where there is a scarcity of pharmacopoeia-mandated materials, the money to buy them and/or the time or expertise to use them appropriately. We also note that in the context of medicine quality surveys, especially surveys using so-called mystery shopper techniques, large numbers of units (for example, tablets) per sample are sometimes hard to acquire. This issue sometimes leads researchers to use fewer units than prescribed by the relevant monograph.

Yet, these modifications are not routinely reported. We urge the medicine quality research community to report their methods in greater detail and to share the results of any modifications (including those adjustments, such as our first modification, that do not work well). Such an evidence base could eventually be integrated into existing standards and guidance to provide feasible and affordable alternative methods for testing the quality of medicines for use in activities such as surveillance where patient safety is not at immediate risk.
